# Chalepin and Chalepensin: Occurrence, Biosynthesis and Therapeutic Potential

**DOI:** 10.3390/molecules26061609

**Published:** 2021-03-14

**Authors:** Lutfun Nahar, Shaymaa Al-Majmaie, Afaf Al-Groshi, Azhar Rasul, Satyajit D. Sarker

**Affiliations:** 1 Laboratory of Growth Regulators, Institute of Experimental Botany ASCR and Palacký University, Šlechtitelů 27, 78371 Olomouc, Czech Republic; 2 Centre for Natural Products Discovery, School of Pharmacy and Biomolecular Sciences, Liverpool John Moores University, James Parsons Building, Byrom Street, Liverpool L3 3AF, UK; bio.shaymaa@yahoo.com (S.A.-M.); afaf.gerushi@gmail.com (A.A.-G.); 3 Cell and Molecular Biology Lab, Department of Zoology, Government College University, Faisalabad 38000, Pakistan; drazharrasul@gmail.com

**Keywords:** *Ruta chalepensis*, Rutaceae, chalepin, chalepensin, bioactivity, biosynthesis

## Abstract

Dihydrofuranocoumarin, chalepin (**1**) and furanocoumarin, chalepensin (**2**) are 3-prenylated bioactive coumarins, first isolated from the well-known medicinal plant *Ruta chalepensis* L. (Fam: Rutaceae) but also distributed in various species of the genera *Boenminghausenia*, *Clausena* and *Ruta*. The distribution of these compounds appears to be restricted to the plants of the family Rutaceae. To date, there have been a considerable number of bioactivity studies performed on coumarins **1** and **2**, which include their anticancer, antidiabetic, antifertility, antimicrobial, antiplatelet aggregation, antiprotozoal, antiviral and calcium antagonistic properties. This review article presents a critical appraisal of publications on bioactivity of these 3-prenylated coumarins in the light of their feasibility as novel therapeutic agents and investigate their natural distribution in the plant kingdom, as well as a plausible biosynthetic route.

## 1. Introduction

**Figure 1:**
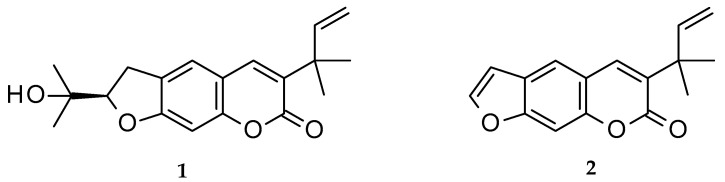
Structures of chalepin (**1**) and chalepensin (**2**).

Chalepin (**1**; mol formula: C_19_H_22_O_4_; mol weight 314) and chalepensin (**2**; mol formula: C_16_H_14_O_3_; mol weight 254) (Figure 1) are, respectively, a dihydrofuranocoumarin and a furanocoumarin, with a prenylation at C-3 of the coumarin core structure. These coumarins, as the names imply, were first isolated from *Ruta chalepensis* L. (Fam: Rutaceae), but are also found in other *Ruta* species, e.g., *R. angustifolia* and a few other plants of the genus *Clausena* (Fam: Rutaceae), e.g., *Clausena anisata* (Willd.) Hook. F. ex Benth. [1,2,3,4]. While chalepin (**1**), also known as heliettin, is optically active, chalepensin (**2**), also known as xylotenin, does not possess any optical activity. Although these coumarins are rather rare in the sense that there are not many 3-prenylated naturally occurring furanocoumarins reported to date, there are quite a good number of bioactivity studies carried out on these compounds. The present review critically appraises publications on bioactivity of these 3-prenylated furanocoumarins in the light of their feasibility as novel therapeutic agents and covers their natural distribution in the plant kingdom, as well as a plausible biosynthetic route.

## 2. Distribution

First isolated from *Ruta chalepensis* more than half a century ago, chalepin (**1**) and chalepensin (**2**) have been further reported mainly from various species of the genera *Clausena* and *Ruta* of the family Rutaceae [4,5]. It appears that these compounds exclusively occur in the family Rutaceae [1,2,3,4,5,6,7,8,9,10,11,12,13,14,15,16,17,18,19,20], and predominantly within these two genera. However, *Boenminghausenia albiflora* var. *japonica (Hook.)* Rchb. Ex Meisn and *B. sessilicarpa* H. Lev. also produce chalepensin (**2**) [6,20] and this genus is phylogenetically close to the genus *Ruta* [21]. Chalepensin (**2**) was further found in the leaves of *Esenbeckia alata* (Karst and Triana) Tr. and Pl. [9], while *E. grandiflora* Mart. was reported to produce chalepin (**1**) [10]. Interestingly, the genus *Esenbeckia* Kunth. is a part of a small group of phylogenetically distant Rutaceae including the genera *Clausena* and *Ruta,* where 3-prenylated coumarins like **1** and **2** are generally produced [9]. Thus, co-occurrence of these 3-prenylated furanocoumarins in these genera might have some chemotaxonomic implications, at least at the family level, within the family Rutaceae. The distribution of these two coumarins (**1** and **2**) is summarized in Table 1. Within the source plants these compounds are well distributed almost in all parts, leaves, stem, flowers and fruits. Although not chalepensin (**2**) itself, a series of 5-*O*-prenylated chalepensin derivatives were reported from *Dorstenia foetida* Schweinf., a medicinal plant from the family Moraceae, distributed in various countries in the Middle-East Asia [22].

## 3. Biosynthesis

**Figure 2:**
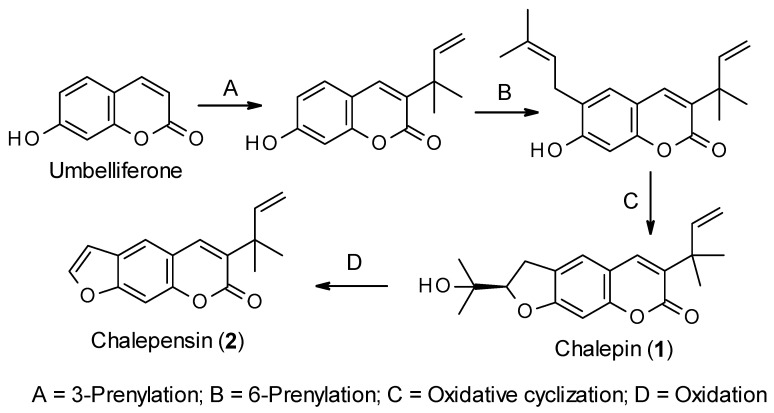
Putative biosynthetic route for the formation of chalepin (**1**) and chalepensin (**2**).

Like all other coumarins, the biosynthesis of chalepin (**1**) and chalepensin (**2**) begins from the simple coumarin umbelliferone, which is formed from the amino acid L-phenylalanine through the formation of *trans*-cinnamic acid, *p*-coumaric acid, 2-hydroxy-*p*-coumaric acid, 2-glucosyloxy-*p*-coumaric acid and 2-glucosyloxy-*p*-*cis*-coumaric acid aided by different enzymes, e.g., cinnamate 4-hydroxylase and 4-coumarate-CoA ligase, 4-coumaroyl 2′-hydroxylase (Figure 2) [23,24]. Sharma et al. [25] studied the biosynthesis of chalepin (**1**) in *Ruta graveolens*. They suggested that 3-(1,1-dimethylallyl)-umbelliferone could be the key intermediate for the biosynthesis of chalepin (**1**), and the dihydrofuran moiety in chalepin (**1**) is formed via prenylation, aided by dimethylallyldiphosphate, at C-6 of the core coumarin skeleton followed by oxidative cyclization with neighboring hydroxyl function at C-7. Generally, prenyltransferases (6-prenyltransferase was identified in *R. graveolens* as a plastidic enzyme) are considered the enzymes involved in the biosynthesis of furano-/dihydrofuranocoumarins through umbelliferone prenylation. Further oxidation of chalepin (**1**) could lead to the formation of the furanocoumarin chalepensin (**2**) in a similar fashion as observed in the conversion of marmesin to psoralen [26]. In fact, biosynthesis of chalepin (**1**) resembles that of 3-prenylated furanocoumarin, rutamarin (acetyl-chalepin) [26]. At this moment, it is not clear from the literature if the prenylation at C-3 takes precedence over that on C-6. In fact, the published information on the biosynthesis of these coumarins **1** and **2** is rather extremely limited, and much work, especially using radioisotopes is much needed to explore other possible routes to the biosynthesis of these compounds.

## 4. Bioactivity

The general, *Clausena* and *Ruta*, the main sources of chalepin (**2**) and chalepensin (**2**), are well known for their uses in traditional medicines, and different studies have established their bioactivities [27,28]. Chalepin (**1**) and chalepensin (**2**) have emerged as two major bioactive components in many of those plants through bioassay-guided isolation protocols, and their bioactivities include antimicrobial, anti-inflammatory, anticancer, antiviral and many more. In this section, using several subsections, a critical appraisal is presented on bioactivities of these two compounds (**1** and **2**) reported in the literature to date (Table 2) [29,30,31,32,33,34,35,36,37,38,39,40,41,42,43,44,45,46,47,48,49,50,51]. Most of the reported bioactivity studies on these compounds involved predominantly in vitro assays and only a handful of in vivo and in silico studies. However, there is no report on any systematic preclinical or clinical trial with these compounds involving human volunteers available in the literature to date.

### 4.1. Antidiabetic Activity

Among the bioactive compounds isolated from the stem bark of *Clausena lansium* (Lour.) Skeels, chalepin (**1**) exhibited antidiabetic properties, exerted through dose-dependent stimulated (glucose-mediated) insulin release in vitro from INS-1 cells (rat insulinoma cell line) [8]. Chalepin (**1**) showed 138% insulin secretory response in vitro at the concentration of 0.1 mg/mL. INS-1 cells are widely used as rat islet β-cell models for screening for antidiabetic properties of plant extracts or purified compounds. They express muscarinic M1 and M3 receptors, which are activated by carbachol to promote insulin release. Chalepensin (**2**) does not appear to have gone through any antidiabetic screening yet. It is known that insulin secretion involves a sequence of events in β-cells that lead to fusion of secretory granules with the plasma membrane; it is secreted primarily in response to glucose, while other nutrients such as free fatty acids and amino acids can augment glucose-induced insulin secretion.

### 4.2. Antifertility Activity

During the assessment of the extracts of *R. chalepensis* var *latifolia* for antifertility activity in rodents, chalepin (**1**) and chalepensin (**2**) were discovered as the major active antifertility principles in the extracts [13]. Despite these compounds showing antifertility activity, most of the tested animals developed cystic and atretic follicles in their ovaries and glomerulocapsular adhesion and segmental fusion in the kidneys. However, no brain toxicity was observed with these compounds. Kong et al. [29] assessed the antifertility activity of the chloroform extracts of the roots, stem and leaves of *R. graveolens* L. in rats and fractionation of the extracts afforded coumarin **2** as the active component with moderate toxicity. Time-dosing experiments showed that this coumarin (**2**) could act at the early stages of pregnancy. The observed antifertility activity of **1** and **2** [13,29] could provide some scientific evidence in support of the traditional uses of *R. chalepensis* as an abortifacient.

### 4.3. Antimicrobial Property

Antimicrobial assay-guided analysis of a root extract of *Clausena anisata* (Willd.) Hook. f. Benth., a well-known medicinal plant used traditionally for the treatment of parasitic infections, influenza, abdominal pain and constipation, afforded chalepin (**1**) as an antibacterial agent, particularly effective against *Bacillus subtilis* with a zone of inhibition of 16 mm as opposed to 15 mm of the positive control cifrofloxacin [4]. This coumarin was also found active against two other pathogenic bacterial strains, *Staphylococcus aureus* and *Pseudomonas aeruginosa*. Chalepensin (**2**), on the other hand, was reported to possess antifungal property and was found to inhibit the growth of the fungal strains, *Candida albicans* and *Cryptococcus neoformans* [30]. However, interestingly, none of these coumarins showed any antimicrobial activity at tested concentrations (50-100 μg/mL) against a range of microorganisms, e.g., *Bacillus subtilis*, *Mycobacterium smegmatis*, *Staphylococcus aureus* and *Candida albicans*, using a modified microtitre-plate assay as reported by El Sayed et al. [52]. Chalepensin (**2**), isolated from *R. chalepensis*, was assessed for antibacterial activity against *Streptococcus mutans* using the method of colony forming units counts in solid medium culture and reduction of tetrazolium salt MTT [3-(4,5-dimethylthiazol-2-yl)-2,5-diphenyltetrazolium bromide] in liquid medium [31] and was shown to significantly inhibit the growth of this bacterial strain with an MIC (minimum inhibitory concentration) of 7.8 μg/mL.

In the most recent study [32] on anti-MRSA (methicillin-resistant *Staphylococcus aureus*) activity of several compounds, mainly coumarins and flavonoids, isolated from *R. chalepensis* grown in Iraq, both chalepin (**2**) and chalepensin (**3**) showed significant antimicrobial activities against the MRSA strains, ATCC 25923, SA-1199B, XU212, MRSA-274819 and EMRSA-15 with MIC values ranging between 32 and 128 µg/mL. In that study, two other furanocoumarins, bergapten and isopimpineline, which do not have a 3-prenylation as in **1** and **2**, were found inactive at tested concentrations. Based on this finding, it was suggested that the prenylation at C-3 of the coumarin nucleus might be a key determinant of anti-MRSA activity. Chalepensin (**2**) was found to be more active than chalepin (**1**) and was subjected to in silico studies to gain an insight into the extent at which this compound (**2**) is able to bind to MRSA proteins and also their drug−like physicochemical characters. In silico studies on compound **2** showed that this compound could have high GI absorption and no violation of the Lipinski rules. It was also shown that chalepensin (**2**) could bind with certain MRSA protein targets, predominantly through hydrogen bonding as well as van de Waals forces. It was suggested that this coumarin could be utilized as a structural template for generating structural analogs and developing potential anti-MRSA therapeutic agents.

### 4.4. Antiprotozoal Activity

One of the major traditional medicinal uses of *R. chalepensis* and other *Ruta* species is their efficacy as antiparasitic agents [28], particularly as an anthelmintic medication. This traditional medicinal use of *R. chalepensis* has prompted antiparasitic activity screening of its extracts and isolated major compounds, including chalepin (**1**) and chalepensin (**2**). Antiprotozoal activity of chalepin (**1**) and chalepensin (**2**), obtained from *R. chalepensis* following a bioassay-guided protocol, against *Entamoeba histolytica*, which is a causative organism of ameoebiasis, was reported as a meeting presentation, but no further full scientific report was published [33]. Both coumarins showed >90% growth inhibition against *E. histolytica*, an anaerobic parasitic amoebozoan, at a concentration of 150 μg/mL with IC_50_ values of 28.67 and 38.71 μg/mL, respectively, for compounds **1** and **2**. However, in a previous study [5], conducted by the same group, evaluated antiprotozoal activity of plants used in northwest Mexican traditional medicine, particularly *Lippia graveolens* Kunth. and *R. chalepensis*, against *E. histolytica,* and chalepensin (**2**) was found to be the main antiprotozoal component in *R. chalepensis*. Earlier, Kundu and Roy [34] carried out in silico studies involving chalepin (**1**) and glyceraldehyde-3-phosphate dehydrogenase (GAPDH) of the pathogenic protozoa *E. histolytica.* It can be noted that GAPDH is a major glycolytic enzyme (~37 kDA), which catalyzes the sixth step of glycolysis, and an attractive drug target like *E. histolytica* lacks a functional citric acid cycle and exclusively depends on glycolysis for its energy needs. Chalepin (**1**) was predicted as a GAPDH inhibitor and structural modifications offering additional polar interactions were suggested to improve potency.

*Trypanosoma cruzi*, a species of parasitic euglenoids, characteristically can bore tissue in another organism and feed on blood and lymph, causing diseases like Chagas disease (also known as American trypanosomiasis) in humans, that affects more than 7 million people worldwide, with Latin American countries being most affected. In recent years, a renewed interest has been observed in the search for antitrypanosomal natural products, especially from higher plants. In many in vitro as well as in silico studies, glycosomal glyceraldehyde-3-phosphate dehydrogenase (gGAPDH) from *T. cruzi* has been used as a target molecule for screening compounds for potential antitrypanosomal activity [35]. In an in silico study with various natural products, chalepin (**1**) emerged as a hit molecule for antitrypanosomal drug discovery [36], and subsequently, a series of 3-piperonylcoumarins were synthesized and tested for their inhibitory activity against gGAPDH. Chalepin (**1**) was shown in silico to possess the highest binding affinity to gGAPDH (IC_50_ = 55.5 μM) among the natural coumarins screened and the best inhibitor of gGAPDH [36,37]. Earlier, during an in vitro screening of natural coumarins for trypanocidal or antitrypanosomal activity, chalepin (**1**) was found to be the most active coumarin with an IC_50_ value of 64 μM [38]. However, to the best of our knowledge, there is no report available to date on antitrypanosomal property of chalepensin (**2**).

### 4.5. Antiviral Activity

Chalepin (**1**), isolated from *R. graveolens*, along with its 28 synthetic analogs were tested for their inhibitory activity on the Epstein–Barr virus (EBV, also known as human herpes virus 4) lytic replication activity [41]. It was noted that most of the synthesized analogs were more active than their parent or precursor, (-)-chalepin (**1**). EVP is a human gamma-herpes virus that infects more than 90% of the human population globally, and preferentially infects B lymphocytes and epithelial cells causing various diseases like Hodgkin’s disease, Burkitt’s lymphoma, nasopharyngeal carcinoma and gastric carcinoma in humans. Thus, inhibition of EBV lytic replication is considered as one of the pragmatic strategies for the treatment of some these diseases.

Chalepin (**1**), isolated from the leaves of *R. angustifolia*, displayed significant inhibitory activity (IC_50_ = 1.7 μg/mL) against hepatitis C virus replication and was found to be more potent than the positive control ribavirin (IC_50_ = 2.8 μg/mL), a well-known antiviral drug used for the treatment of hepatitis C and other viral diseases [39]. In continuation of their study, they have recently reported enhancement of antihepatitis C virus activity of chalepin (**1**) in combination with conventional antiviral drugs including cyclosporine A, daclatasvir, ribavirin, simeprevir and telaprevir [40]. It was found that chalepin (**1**) could enhance antihepatitis C activities of these conventional drugs with a synergistic combination index of <1. It could be considered as an excellent finding as the need for new and effective drugs for treating hepatitis C is of paramount importance. It can be mentioned that hepatitis C virus infects around 71 million people globally, causes severe liver disease, e.g., liver cancer and deaths; the WHO (World Health Organization) estimated that in 2016, about 400,000 people died from hepatitis C, mainly from liver cirrhosis and liver cancer [53,54].

Like many other antiviral coumarins including some 3-substitued ones, it can be assumed that chalepin (**1**) might offer antiviral activity through inhibition of various proteins that are involved in the transcription/translation processes essential for viral life cycle at different stages, and via modulation of host cell signaling, NF-kB (nuclear factor κB), and inflammatory redox-sensitive pathways and thus blocking viral replication [54]. However, clearly, further research is necessary to understand and establish definite mode of antiviral action mechanism of chalepin (**1**). However, there is no data available on any antiviral property of chalepensin (**2**) to date.

### 4.6. Cytotoxicity (Potential Anticancer and Antitumor Activity)

Cancer is one of the major causes of human mortality and morbidity. Currently available cancer treatment options or modalities are rather limited, and often suffer from severe side effects. Therefore, the search for new, effective, safe and affordable anticancer drugs is a part of many major modern drug discovery initiatives worldwide. Natural products have long been considered one of the major contributors in the continuing search for new anticancer molecules for safer and more effective anticancer drug development, and evidently, have already provided several successful anticancer drugs, e.g., taxol, vincristine and vinblastine [55]. The most common starting point in the search for anticancer molecules is the screening compounds for cytotoxicity against various human cancer cell lines because cytotoxicity is regarded as one of the major characteristics of anticancer agents. In order to assess anticancer potential of chalepin (**1**) and chalepensin (**2**), cytotoxicity of these compounds has been assessed against different human cancer cell lines in vitro, and some mechanistic studies on how they kill the cancer cells have also been published, showing anticancer and antitumor potential of these compounds (Table 2).

Chalepin (**1**), isolated from *Clausena emarginata* C. C. Huang, has been found to possess significant cytotoxicity against five human cancer cell lines including human leukemia (HL-60), hepatocarcinoma (SMMC-7721), lung carcinoma (A-549), breast cancer (MCF-7) and colon adenocarcinoma (SW-480) with IC_50_ values comparable to that of the positive control, doxorubicin [7]. Chalepin (**1**), isolated from *Ruta angustifolia* Pers., was demonstrated to induce apoptosis through phosphatidylserine externalizations and DNA fragmentation in breast cancer cell line, MCF-7 [12,42]; this compound was considerably cytotoxic to MCF-7 cells, moderately cytotoxic to the epithelial human breast cancer cells (MDA-MB231), but not cytotoxic to normal cells, MRC-5 (Medical Research Council cell strain 5) in the SRB (sulforhodamine B) assay [56]. MRC-5 is a diploid cell culture line comprising fibroblasts, first developed from the lung tissue of a 14-week-old aborted Caucasian male fetus. It can be mentioned here that apoptosis is a process by which cell commit suicide and is eliminated from the system; induction of apoptosis, a cell toxicity pathway, is considered as one of the early-stage mechanism for compounds to exert anticancer activity. This differential cytotoxicity against cancer cells and noncancerous cells might make this compound an ideal candidate, or at least a structural template, for anticancer drug development.

Earlier, in order to understand how chalepin (**1**) could exert its anticancer potential, a study conducted by Richardson et al. [11], revealed that this compound could dose-dependently exhibit cell cycle arrest at S phase, suppress nuclear factor kappa B (NFκB) pathway, signal transducer and activation of transcription 3 phosphorylation and extrinsic apoptotic pathway in human non-small cell lung cancer cell line A-549. Cell cycle analysis using the flow cytometry confirmed that chalepin (**1**) could inhibit cell cycle at S phase (synthesis phase), which is the phase of the cell cycle, where DNA is replicated and occurs between the G_1_ and G_2_ phases. Since accurate duplication of the genome is essential for successful cell division to take place, the processes involved in the S phase are tightly regulated and widely conserved. A significant accumulation of cells in the S phase was observed after chalepin (**1**) treatment (45 μg/mL) for 48 (accumulation 27.7%) and 72 h (accumulation 25.4%), whereas the accumulation was only about 4% for the untreated cells [11]. It is well known that there is a remarkable link between cell cycle and cancer, as cell cycle appears to be the machinery that controls cell proliferation, and uncontrolled cell proliferation happens in cancer. The suppression of the NF-κB pathway by chalepin (**1**) was shown to be through modulation of the p65 subunit of NF-κB, where the phosphorylation of p65 and the translocation of the p65 subunit to nucleus were inhibited [11]. It can be noted that the NF-κB pathway is generally induced by carcinogens and inflammatory agents. Thus, suppression of NF-κB pathway by chalepin (**1**) could suggest its potential as an anticancer agent.

Caspase 8 is implicated to the activation of the intrinsic apoptotic pathway, and enhancement of caspase 8 activity can be exploited to identify compounds with plausible anticancer activity. In chalepin (**1**) treated cells, a significantly increased level of caspase 8 activity was noticed, when compared to the control; after 48 and 72 h of incubations, chalepin (**1**) (45 μg/mL) enhanced caspase 8 activity, respectively, by 5-fold and 8.6-fold [11].

This group of researchers also demonstrated that chalepin (**1**) and chalepensin (**2**) could induce mitochondrial mediated apoptosis in lung carcinoma cells (A-549), with chalepin (**1**) being more cytotoxic than chalepensin (**2**) [3]; chalepin (**1**) exhibited selective cytotoxicity against A-549 cells with an IC_50_ value of 8.69 μg/mL (27.64 μM). Chalepin (**1**) was mildly toxic to the normal cell line with an IC_50_ value of 23.4 μg/mL. Chalepensin (**2**) exhibited considerable cytotoxic property against A-549 cell line with IC_50_ value of 18.5 μg/mL, while the cytotoxicity (IC_50_ = 23.4 μg/mL) of this coumarin against noncancerous MRC-5 human lung fibroblast cell line was of moderate level as was with chalepin (**1**). Chalepin (**1**) showed morphological changes, typical for apoptosis, e.g., plasma membrane blebbing, cell vacuolization, echinoid spiking, chromatin condensation, formation of apoptotic bodies, cell shrinkage and nuclear fragmentation. Both coumarins (**1** and **2**) were found to downregulate inhibitors of apoptosis such as Bcl-2, survivin, Bcl-xl and cFLIP. They also triggered release of cytochrome c and activated caspases 9 and 3 to induce apoptosis. Chalepensin (**2**) was shown to possess cytotoxicity against colon (H-T29), lung (A-549), breast (MCF-7), kidney (A-498), and pancreatic (PACA-2) cancer cell lines [3].

Wu et al. [17] screened 19 compounds isolated from *Ruta chalepensis*, including chalepensin (**2**), for their potential cytotoxicity against KB (keratin forming tumor), Hela, DLD (colorectal adenocarcinoma) and Hepa tumor cell lines, but chalepensin (**2**) was found to be inactive against any of these cell lines at tested concentrations. From the available literature data, it is obvious that chalepin (**1**) is more cytotoxic than chalepensin (**2**). However, considerably more work has been carried out with chalepin (**1**) than with chalepensin (**2**) to date, and further comparative work may be necessary to gain a better insight into their anticancer potential.

### 4.7. Miscellaneous Activities

Spasmolytic activities of chalepin (**1**) and a few other coumarins, isolated from *Boenninghausenia albiflora* (Hook.) Rchb. Ex Meisn., were reported by Rizvi et al. [43]. Effects of aqueous extracts of *R. graveolens* and its ingredients, chalepensin (**2**) being one of them, on major drug metabolizing enzymes, cytochrome P450, uridine diphosphate (UDP)-glucuronosyltransferase and reduced nicotinamide adenine dinucleotide (phosphate) (NAD(P)H)-quinone oxidoreductase, were evaluated in mice [15]. The repeated administration of *R. graveolens* extract, rich in rutin and chalepensin (**2**), could induce hepatic CYP1a and CYP2b activities in a dose-dependent fashion. It was observed that male mice were more responsive than female mice to the extract-medicated induction of UGT (uridine glucuronosyltransferase). Earlier, the same group of researchers [48] showed mechanism-based inhibition of CYP1a1 and CYP3A4 by chalepensin (**2**), while this compound was also found to inhibit human CYP1a2, CYP2a13, CYP2c9, CYP2d6 and CYP2e1.

In order to study the in vivo effect of chalepensin (**2**), Lo et al. [44] assessed its effect on multiple hepatic P450 enzymes in C57BL/6JNarl mice, and observed that this coumarin, after oral administration (10 mg/kg) in mice for 7 days, could decrease hepatic coumarin 7-hydroxylation by CYP2a, and increase 7-pentoxyresorufin *O*-dealkylation by CYP2b, without affecting the activities of other CYP enzymes. It was further observed that the suicidal inhibition of CYP2a5 and the constitutive androstane receptor (CAR) mediated CYP2b9/10 induction simultaneously happened in chalepensin (**2**)-treated mice. Previously, Ueng et al. [46,47] and Lo et al. [45] carried out related extensive studies on mechanism-based inhibition of CYP enzymes by chalepensin (**2**) in various in vitro and in vivo models. However, there is no report on such activities of chalepin (**1**) available in the published literature to date.

In a study conducted by Wu et al. [17], chalepensin (**2**) at 100 μg/mL concentration displayed significant antiplatelet aggregation activity, induced by arachidonic acid and collagen. This coumarin, isolated from *Boenninghausenia albiflora* var*. japonica,* was also reported to possess calcium antagonistic property [6].

## 5. Mutagenicity and Other Toxicities

The mutagenicity of chalepin (**1**) was assessed at the HGPRT locus (AzGr) in Chinese hamster V79 cells [50], and this compound was found to be mutagenic. Chalepin (**1**), isolated from *Clausena aniseta*, a well-known medicinal plant from West Africa, showed anticoagulant (blood-thinning) activity when administered to rats in a single dose [51], and it could depress aniline hydroxylase activity. Ethylmorphine demethylase, hepatic DNA, reduced glutathione and glucose-6-phosphatase were unaffected by chalepin (**1**) treatment at a dose of 50 mg/kg for 3 days prior to sacrifice. This coumarin also resulted in α-1-globulin increase and a decrease in β-globulin content of the serum. Intraperitoneal treatment with chalepin (100 mg/kg) for 2 days caused death of 4 rats out of 100 within 48 h of treatment. Livers of dead rats showed generalized necrosis of hepatocytes. Chalepin (**1**) induced alterations in the serum protein pattern within this period. Liver lesions were observed in chalepin treated animals and were characterized by mild necrosis of hepatocytes. However, no report on mutagenicity of chalepensin (**2**) is available to date.

## 6. Drugability’ of Chalepin (1) and Chalepensin (2)

“Drugability” can simply be defined as the ability of a compound to be used as a pharmaceutical drug. In order for a molecule to be developed as a drug, it must have certain physicochemical characteristics, which can be measured or predicted by various experimental or mathematical models. The Lipinski rule of five, formulated in 1997 by Christopher A. Lipniski, can be used, albeit not conclusively, to predict whether a compound could be an ideal candidate as a drug molecule, i.e., whether a compound possesses “druglikeness” or not [57]. This rule states that an orally active drug does not have more than one violation of the following criteria: a molecular mass less than 500 Daltons, no more than five hydrogen donors, no more than 10 hydrogen bonds and an octanol-water partition coefficient (log *P*) that does not exceed five. Sometimes an additional criterion, “molar refractivity should be between 40–130” is also added to the above rule. If we consider these criteria in relation to chalepin (**1**) and chalepensin (**2**), both compounds tend to follow Lipinski rule of five, and there is no violation of this rule whatsoever (Table 3), which suggests that these compounds possess “druglikeness” or “drugability” and have the potential for further development as commercial drugs. However, it must be noted that this rule of five was originally presented to aid the development of orally bioavailable drugs and was not intended for guiding the medicinal chemistry in the development of all small-molecule drugs. Moreover, there is hardly any reliable experimental bioavailability data available on these coumarins (**1** and **2**) to make any connections between bioavailability and the predicted values for the criteria shown in Table 3.

## 7. Conclusions

The present work generated the first comprehensive and critical review of published literature on chalepin (**1**) and chalepensin (**2**), revealing various bioactivities of these compounds and their potential as new therapeutic agents. Among the activities, it appeared that antiprotozoal, antiviral and particularly anticancer activities bear promises for these compounds for further consideration for development as therapeutic agents, when considered in the light of nonviolation of the Lipinski rule of five and low level of toxicities. However, there is no report on any systematic preclinical or clinical trial with these compounds involving human volunteers available in the literature to date. Therefore, further studies, including controlled preclinical and clinical trials, are still needed before we can comment on the true therapeutic potential of these compounds.

## Figures and Tables

**Table 1:** Distribution of chalepin (**1**) and chalepensin (**2**) in the plant kingdom.

Plant Names	Family	Chalepin (1)	Chalepensin (2)	References
*Boenminghausenia albiflora* var. *japonica* (Hook.) Rchb. Ex Meisn.	Rutaceae	−	+	[6]
*Boenminghausenia sessilicarpa* H. Lev.	Rutaceae	−	+	[20]
*Clausena anisata* (Willd.) Hook. F. ex Benth.	Rutaceae	+	−	[4,7]
*Clausena emarginata* C. C. Huang	Rutaceae	+	−	[4,7]
*Clausena indica* (Dalz.) Oliver	Rutaceae	+	+	[1]
*Clausena lansium* (Lour.) Skeels	Rutaceae	+	+	[8]
*Esenbeckia alata* (Karst & Triana) Tr. & Pl.	Rutaceae	−	+	[9]
*Esenbeckia grandiflora* Mart.	Rutaceae	+	−	[10]
*Ruta angustifolia* L. Pers	Rutaceae	+	−	[3,11,12]
*Ruta chalepensis* L.	Rutaceae	+	+	[5,13,14,15]
*Ruta graveolens* L.	Rutaceae	+	+	[16,17]
*Ruta montana* L.	Rutaceae	−	+	[18]
*Stauranthus perforatus* Liebm.	Rutaceae	+	+	[19]

+ = Found; − = Not found.

**Table 2:** Reported bioactivities of chalepin (**1**) and chalepensin (**2**).

Bioactivity	Chalepin (1)	Chalepensin (2)	References
Antidiabetic	+	NR	[8]
Antifertility	+	+	[13,29]
Antimicrobial	+	+	[4,30,31,32]
Antiplatelet aggregation	NR	+	[17]
Antiprotozoal	+	+	[5,33,34,35,36,37,38]
Antiviral	+	NR	[39,40,41]
Calcium antagonist	NR	+	[6]
Cytotoxicity (potential anticancer and antitumor)	+	+	[3,7,11,12,42]
Spasmolytic	+	NR	[43]
Effect on drug metabolizing enzymes	NR	+	[44,45,46,47,48,49]
Mutagenicity and other toxicities	+	NR	[50,51]

NR = No report available; + = Active.

**Table 3:** **“**Druglikeness” of chalepin (**1**) and chalepensin (**2**) *.

Criteria	Chalepin (1)	Chalepensin (2)
Molar mass	314	254
Hydrogen bond donor	1	0
Hydrogen bond acceptors	4	3
Log *P*	3.72	4.32
Molar refractivity	86.6 cm^3^	72.5 cm^3^
Lipinski rule of 5 violation	0	0

* Data obtained from ChemSpider (www.chemspider.com, (accessed on 24 February 2021)) and DrugBank (https://go.drugbank.com/drugs/DB02205, (accessed on 24 February 2021)).

## Data Availability

All relevant data have been presented as an integral part of this man.
